# Characterization of Nonsmall Cell Lung Carcinoma in Limited Biopsy Samples and Identifying Optimal Immunohistochemical Marker Combinations in Resource-Constrained Setup: An Institutional Experience

**DOI:** 10.1055/s-0044-1791560

**Published:** 2024-10-11

**Authors:** Ankita Grover, Md Ali Osama, Shashi Dhawan

**Affiliations:** 1 Department of Pathology, Goyal Hospital and Research Centre Pvt. Ltd, Jodhpur, Rajasthan, India; 2 Department of Pathology, Lady Hardinge Medical College, New Delhi, India; 3 Department of Histopathology, Sir Gangaram Hospital, New Delhi, India

**Keywords:** lung, immunohistochemistry, nonsmall cell, squamous, adenocarcinoma

## Abstract

**Background** The incorporation of immunohistochemical markers in the analysis of small biopsy samples, as outlined in the fourth edition of the World Health Organization Blue books, represents a noteworthy advancement in the diagnosis of advanced-stage lung carcinoma. This improved the histological classification for poorly differentiated nonsmall cell lung carcinomas (NSCLCs), especially in small biopsy specimens. Despite challenges in obtaining viable cells from diminutive tumor samples, a focused immunohistochemical panel effectively distinguishes histological types in most NSCLC. This preserves tissue for subsequent molecular testing.

**Material and Methods** This study examined 130 consecutive lung biopsy cases initially diagnosed as NSCLC, including various biopsy types (transbronchial, endobronchial, ultrasound-guided, computed tomography-guided). Carcinomas were categorized based on specific characteristics, such as glands and/or mucin for adenocarcinomas, keratinization and/or intercellular bridges for squamous cell carcinomas, and recognition of poorly differentiated NSCLC. Cases lacking clear morphological attributes underwent reclassification using immunohistochemical markers (TTF1, Napsin A, p63, and p40).

**Results** TTF1 exhibited superior sensitivity (97.56%) and specificity (96.77%) for adenocarcinoma compared with Napsin A, with sensitivity and specificity at 90.24 and 93.3%, respectively. p63 and p40 demonstrated 100% sensitivity for squamous cell carcinoma, with p40 being more specific than p63 (100% vs. 82.92%). Using TTF1 and p63 as a conventional panel, 87% of cases were subtyped. However, the combination of TTF1 and p40 achieved accurate classification in 94.66% (71/75) of cases, and all four markers allowed subtype identification in 97.2% (73/75) of cases.

**Conclusion** In a resource-constrained setting, subtyping NSCLC in small biopsy can be effectively accomplished using a minimal panel consisting of TTF1 and p40 immunohistochemical markers.

## Introduction

Lung cancer, a predominant contributor to cancer-related fatalities globally, is responsible for an estimated 1.8 million deaths each year. 1 Approximately 85% of newly diagnosed lung cancer cases belong to the nonsmall cell type (nonsmall cell lung carcinoma [NSCLC]) category, encompassing adenocarcinomas, squamous cell types, and other less common subtypes. 2 Carcinomas that pose challenges in classification based solely on morphological characteristics are categorized as poorly differentiated. Within this group, approximately 50 to 60% are identified as adenocarcinomas, with squamous cell carcinomas being the subsequent second most common subtype. 2 3 Prior to 2004, there were no therapeutic implications associated with distinguishing between various histological subtypes of lung carcinoma. The only clinically significant distinction lay between small cell carcinoma and nonsmall cell carcinoma. However, the advent of targeted therapies has underscored the necessity for precise histological subtyping. For instance, lung adenocarcinomas are frequently linked to epidermal growth factor receptor (EGFR) mutations and EML4-ALK rearrangements, and they can be effectively treated with tyrosine kinase inhibitors such as gefitinib and crizotinib, respectively. 3 Furthermore, it has been demonstrated that adenocarcinoma patients tend to experience better outcomes compared with those with squamous cell carcinoma when treated with pemetrexed therapy. 4 As novel therapies specifically designed for lung adenocarcinoma and squamous cell carcinoma have emerged, the accurate differentiation between these tumor types has become increasingly crucial. While there is a clear demand for pathologists to accurately subclassify nonsmall cell carcinoma, distinguishing between squamous and adenocarcinoma morphologically in a small biopsy is very challenging. It is well known that the application of immunohistochemical (IHC) markers has led to an increased accuracy in diagnosis and thus, numerous IHC markers have already been explored for their utility in distinguishing pulmonary squamous cell carcinoma and adenocarcinoma. 5 There is growing evidence that immunohistochemistry as an adjunctive tool increases the accuracy and reproducibility of subtyping of poorly differentiated NSCLCs. As the majority of lung cancer is diagnosed on small biopsies or cytology specimens, often obtained by increasingly sophisticated diagnostic procedures, the pathologist must obtain maximum diagnostic yield from these small and valuable tissue samples. These increasingly small diagnostic biopsy and cytology specimens are no longer required purely for confirmation of malignancy and tumor subtyping, but there must be sufficient tumor tissue available for molecular testing to complete the pathological diagnostic assessment. The 2011 International Association for the Study of Lung Cancer (IASLC)/American Thoracic Society/European Respiratory Society guidelines strongly encourage surgical pathologists to minimize the amount of tissue used for diagnosis, in particular by limiting the number of first-line IHC stains. 6 Thus, this study aimed at evaluating the most useful limited IHC panel including TTF1, Napsin A, p63, and p40 for subclassifying NSCLC.

## Material and Methods

### Case Selection

In this research, a total of 130 consecutive cases were enrolled from the Pathology Department, Sir Gangaram Hospital, New Delhi. The study encompassed all lung biopsies, including transbronchial, endobronchial, ultrasound-guided, and computed tomography-guided procedures, that were initially diagnosed as nonsmall cell carcinoma upon examination of hematoxylin and eosin (H&E) stained sections. Excluded from the study were lung biopsies related to neuroendocrine tumors, metastatic tumors, and other nonepithelial tumor types. Additionally, cases with insufficient material for a definitive diagnosis and/or IHC analysis were also excluded from the study.

### Microscopic Examination

Following the biopsy processing, two sections were meticulously prepared and subjected to routine H&E staining. The categorization of carcinomas was performed by evaluating histological characteristics, specifically looking for the presence of glands and/or mucin for adenocarcinomas, keratinization and/or intercellular bridges for squamous cell carcinomas, and identifying poorly differentiated nonsmall cell carcinomas. Instances that did not exhibit distinct morphological features underwent reclassification using IHC markers. The results of IHC markers in well-differentiated cases were considered as the reference or “gold standard” for classification.

### Immunohistochemistry

After analysis of H &E slides, IHC staining for TTF1, Napsin A, p63, and p40 were assessed. IHC was done on 2 to 3 µm thick sections taken on coated slides. IHC staining was performed in fully automated BioGenex Laboratries, Xmatrx. Antibodies included TTF1 (mouse monoclonal antibody, clone-8G7G3/1, Dako, United States, 1:50, prediluted), p63 (mouse monoclonal antibody, clone 4A4, Bio SB, United States, dilutions 1:100), Napsin A (mouse monoclonal antibody, clone IP64, Cell Marque, United States, 1:300 dilutions), p40 (polyclonal rabbit immunoglobulin antibody, clone BC28, Biocare Medical, United States, 1:100–1:1000 dilutions). The scoring of TTF1, p63, and p40 staining was done by recording percentage of nuclear immunoreactive tumor cells. TTF1 is scored as strongly positive (more than 50% tumor cells positive), positive (> 1 to 49% tumor cell showing weak positivity), and negative (< 1% tumor cells positive). p63 and p40 were scored as positive (> 10% of tumor cells positive), focally positive when mostly negative but contained small areas of tumor in which nearly all cells stained positive, and negative when tumor showed less than 10% overall staining and no focal areas of positive staining. Napsin A shows granular cytoplasmic staining and is scored as negative (no staining to minimal light brown to dust), weak positive (minimal, patchy, or diffuse staining), and strong positive (moderate to intense brown, granular, staining). Appropriate controls for each marker were included in all the cases.

### Statistical Analysis

SPSS version 17.0 software was used for the analysis. Categorical variables were expressed as frequencies (%). Chi-square test was used to see the association among various variables. *p* -Values < 0.05 were taken as significant.

## Results

A total of 130 cases of NSCLC was included in the study. The median age at diagnosis was 62 years. Male-to-female ratio was 3.3:1. Based on morphological features, 39% (51/130) cases could be classified on H&E alone. Nineteen cases showed features of keratinization and/or intercellular bridging. Gland formation and/or evidence of mucin production was seen in 30 cases of adenocarcinoma and out of these, 4 cases were mucinous adenocarcinoma. Two cases revealed features of both squamous cell carcinoma and adenocarcinoma in different areas, thus classified as adenosquamous carcinoma. Note that 60.7% (79/130) cases could not be subclassified on H&E alone, hence they were classified on the basis of immunoprofiles. The results of immunoprofiles of well-differentiated group are as shown in Table 1 . Two cases of adenosquamous carcinoma have not been included in the table but the immunoreactivity was in concordance with all the markers. Four cases of adenocarcinoma which were negative for TTF1 staining were mucinous adenocarcinoma on morphology, as Napsin A was strongly positive in all these cases. In instances of squamous cell carcinoma displaying pseudoglandular differentiation in morphology and adenocarcinoma featuring tumor cells arranged in diffuse sheets with squamoid differentiation, particularly within small biopsy samples, the necessity for IHC analysis was paramount. Immunoprofiles obtained for adenocarcinomas and squamous cell carcinomas in well-differentiated group were in total agreement to the general staining characteristics for each subtype. Thus, these cases served as a gold standard for analyzing the poorly differentiated group.

**Table 1:** Sensitivity and specificity of individual in well-differentiated NSCLC, *N* = 49

Marker	ADCA, *N* = 26	SQCCA, *N* = 19	Sensitivity (%)	Specificity (%)	PPV (%)	NPV (%)	*p* -Value
TTF1	26/26	0/19	86.6	100	86.6	82.6	< 0.001
Napsin A	22/26	1/19	84.61	95	84.15	78.2	< 0.004
p63	2/26	19/19	100	92.3	90.47	92.3	< 0.001
p40	0/26	19/19	100	100	100	100	< 0.001

Abbreviations: ADCA, adenocarcinoma; NPV, negative predictive value; NSCLC, nonsmall cell lung carcinoma; PPV, positive predictive value; SQCCA, squamous cell carcinoma.

### Subtyping Based on a Limited IHC Panel

**Fig. 1:**
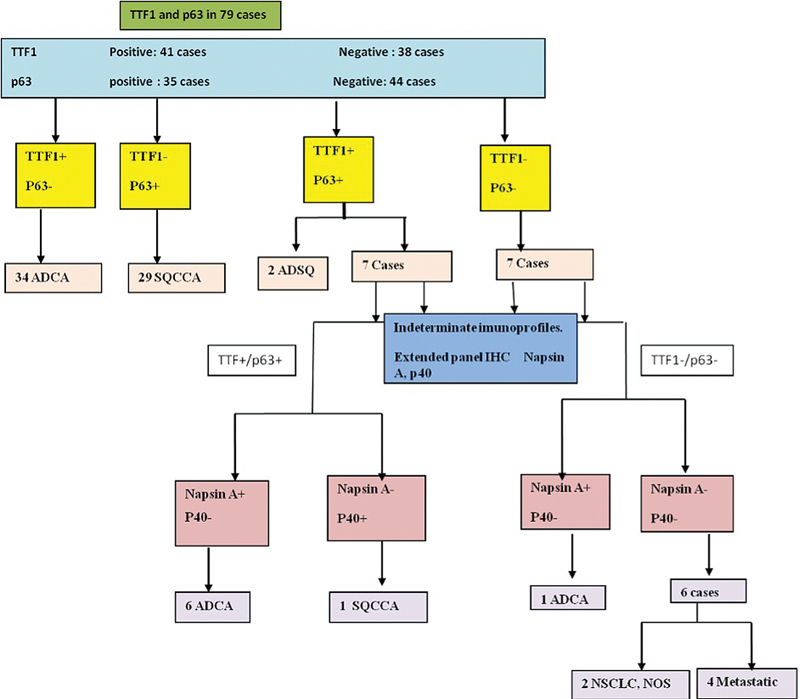
Subtyping of NSCLC, NOS with a limited panel of TTF1 and p63 followed by extended panel using Napsin A and p40 in indeterminate cases. ADCA, adenocarcinoma; SQCCA, squamous cell carcinoma; ADSQ, adenosquamous carcinoma, NSCLC, NOS, nonsmall cell lung carcinoma, not otherwise specified.

TTF1 and p63 are conventional markers for differentiating squamous cell carcinoma and adenocarcinoma. Keeping in view the IASLC recommendation to conserve tissue we attempted a limited panel analysis of poorly differentiated NSCLC ( Table 2 ). Among the 79 poorly differentiated NSCLC cases in the first step, various permutations and combinations using IHC panel (TTF1, Napsin A, p63, and p40) were analyzed as shown in Fig. 1 .

**Table 2:** Immunoprofiles of poorly differentiated NSCLC, *N* = 79

TTF1	Napsin A	P63	P40	No. of cases	Final
+	+	−	−	30	ADCA
+	−	−	−	4	ADCA
+	+	Focal+	−	7	ADCA
−	+	−	−	1	ADCA
−	−	+	+	29	SQCCA
Weak+	−	+	+	1	SQCCA
+	+	+	+	2	ADSQ [diff. areas]
−	−	−	−	6	NSCLC, NOS
−	−	−	−	4	Metastatic [on workup]

Abbreviations: ADCA, adenocarcinoma; ADSQ, adenosquamous carcinoma; NSCLC, NOS, nonsmall cell lung carcinoma, not otherwise specified; SQCCA, squamous cell carcinoma.

**Fig. 2:**
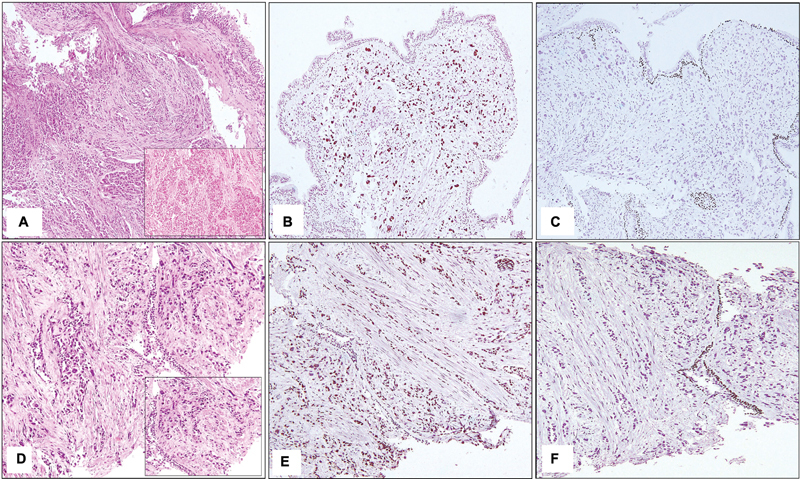
( **A** ) Poorly differentiated tumor lacking features of differentiation (hematoxylin and eosin [H&E] 200×) [Inset - Tumor cells present in sheets and nests, showing marked pleomorphism (H&E 400×)]. ( **B** ) TTF1 positivity in tumor cells (200×). ( **C** ) p40 is negative in tumor cells. Overlying bronchial epithelium and entrapped epithelium showing nuclear positivity serving as internal control (200×). ( **D** ) Tumor cells present in cords and singly lacking any morphological differentiation in a desmoplastic stroma (H&E 200×) [Inset - Markedly pleomorphic tumor cells lacking any feature of differentiation (H&E 400×)]. ( **E** ) TTF1 showing nuclear staining in tumor cells (200×). ( **F** ) p63 negative in tumor cells. Overlying bronchial epithelium showing nuclear positivity serves as an internal control (200×).

**Fig. 3:**
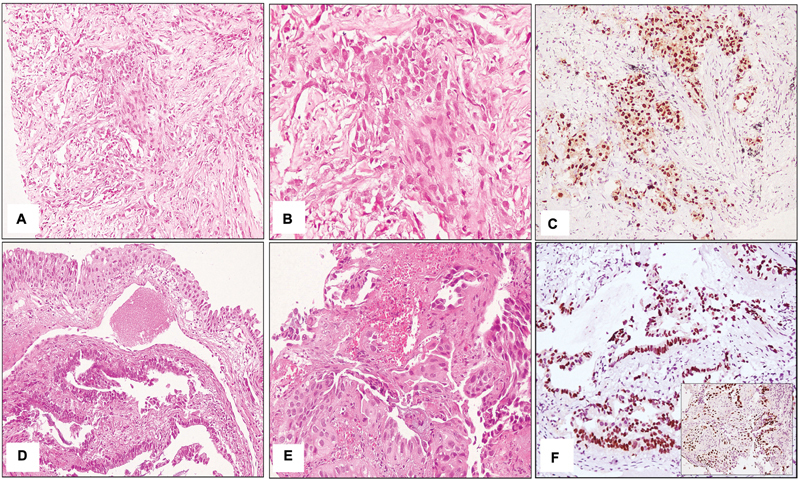
( **A** ) Tumor cells present in nests lacking any morphological differentiation (hematoxylin and eosin (H&E] 200×). ( **B** ) Pleomorphic tumor cells in a desmoplastic stroma, H&E 400× . ( **C** ) p63 showing nuclear immunoreactivity in tumor cells (400×). ( **D** ) Areas of glandular differentiation seen in the infiltrating tumor present in the bronchial wall. Overlying bronchial epithelium appears dysplastic (H&E 200×). ( **E** ) Areas of squamous differentiation represented by tumor nests with intercellular bridges and single cell keratinization (H&E 400×). ( **F** ) TTF1 positivity in areas showing glandular differentiation while negative in tumor cells showing squamous differentiation (400×) [Inset - p63 positivity in areas showing squamous differentiation of the tumor (400×)].

TTF1 +/p63 **–** immunoprofile which was in concordance with a diagnosis of adenocarcinoma was obtained in 34 cases ( Fig. 2A–F )
TTF1-/p63 **+** immunoprofile which is a classical squamous cell carcinoma immunoprofile was found in 29 cases ( Fig. 3A–C )

In two cases both TTF1 and p63 were expressed in different areas, thus these cases were classified as adenosquamous carcinoma ( Fig. 3D–F ).

**Fig. 4:**
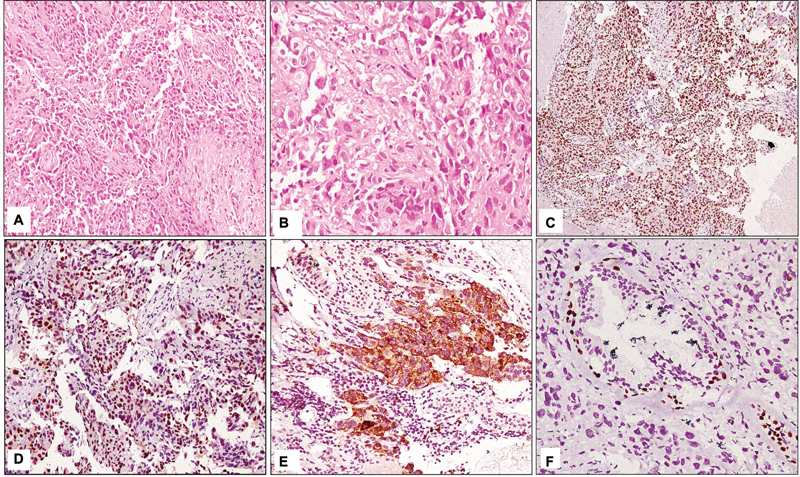
( **A** ) Tumor cells present in sheets and nests with no features of differentiation (hematoxylin and eosin [H&E] 200×). ( **B** ) Moderately pleomorphic tumor cells with vesicular chromatin and small nucleoli (H&E 400×). ( **C** ) Tumor cells showing positivity for TTF1 immunostain (200×). ( **D** ) Tumor cells showing aberrant positive staining with p63 (200×). ( **E** ) Cytoplasmic positivity of Napsin A immunostain in tumor cells (400×). ( **F** ) p40 completely negative in tumor cells. Internal control positive in myoepithelial cells (400×).

In seven cases, TTF1 and p63 were coexpressed in the same tumor cells. In these cases, p63 stained 25 to 75% tumor cells while TTF1 staining pattern ranged from 10 to 80% tumor cells. Seven cases were double negative. Thus, these 14 cases with indeterminate immunoprofiles were subjected to an extended panel of IHC comprising Napsin A and p40. Out of them seven cases which coexpressed TTF1 and p63, six cases were Napsin A positive and p40 negative ( Fig. 4A–F ). One case was positive for p40 and negative for Napsin A. Among the seven double-negative cases, one case was positive for Napsin A while all the seven cases were negative for p40. Thus, with an extended panel seven cases were classified as adenocarcinoma and one case was classified as squamous cell carcinoma. Six were negative for all the four markers. On extensive radiological and biochemical evaluation, four cases were found to have a primary tumor at other site. Hence, two cases were finally classified as NSCLC, not otherwise specified since these cases lacked any feature of morphological or IHC differentiation. Synaptophysin was also performed to exclude large cell neuroendocrine carcinoma, but was negative.

The individual sensitivity and specificity of these markers is shown in Table 3 . Two cases of adenosquamous carcinoma have been excluded from sensitivity and specificity of individual markers. TTF1 had a higher sensitivity and specificity than Napsin A. Both p63 and p40 were equally sensitive for squamous cell carcinoma. However, p40 was more specific than p63.

**Table 3:** Sensitivity and specificity of individual in poorly differentiated NSCLC, *N* = 71

IHC marker	ADCA	SQCCA	Sensitivity	Specificity	PPV	NPV	*p* -Value
TTF1			97.56%	96.77%	97.5%	96.77%	< 0.001
Positive Negative	40/41 1/41	1/30 29/30					
Napsin			90.24%	81.08%	94.87%	88.23%	< 0.001
Positive Negative	37/41 4/41	2/30 28/30					
p63			100%	82.92%	81.08%	100%	< 0.001
Positive Negative	7/41 34/41	30/30 0/30					
p40			100%	100%	100%	100%	< 0.001
Positive Negative	0/41 41/41	30/30 0/41					

Abbreviations: ADCA, adenocarcinoma; IHC, immunohistochemical; NPV, negative predictive value; NSCLC, nonsmall cell lung carcinoma; PPV, positive predictive value; SQCCA, squamous cell carcinoma.

## Discussion

In the era of targeted therapies and immunotherapy, the primary challenge lies in obtaining sufficient tissue to identify patient subsets most responsive to these personalized approaches. In the past 15 years, remarkable therapeutic advancements have been achieved, notably with EGFR tyrosine kinase inhibitors for tumors harboring EGFR sensitizing mutations. Additionally, breakthroughs include inhibitors targeting ALK, ROS1, BRAF, RET, and NTRK, alongside the recent integration of immune checkpoint pathway inhibitors in both second-line and front-line treatments for NSCLC. 7 It is crucial to highlight that these immunotherapy strategies necessitate the alignment of known antigens or pathways with specialized antibodies. Consequently, the integration of diagnostics and therapeutics stands as a pivotal aspect of immunotherapy. Collecting multiple biopsy samples with the capability for distinct use in IHC staining and molecular testing proves advantageous. The most effective approach differs among institutions, relying on the expertise of local physicians, including pulmonologists, radiologists, surgeons, and cytopathologists, who acquire the specimens. A recent progression involves utilizing cell-free deoxyribonucleic acid (DNA) extracted from plasma as a reservoir of tumor-derived DNA for molecular testing. While this method has demonstrated utility in the noninvasive identification of driver mutations, it is hindered by its limited sensitivity. 8

In this study, out of 130 cases 39.2% (51/130) could be histologically subtyped on H&E alone and 60.7% (79/130) of cases could not be further subtyped on the basis of classical histological features like keratinization, gland formation, or mucin production. The lower percentage of cases diagnosed solely based on H&E staining is likely influenced by several factors. First, our hospital functions as a tertiary care center, which means that we often receive more advanced-stage tumors. Additionally, our classification process adheres to strict World Health Organization criteria for differentiation, which can contribute to a lower rate of diagnosis based solely on H&E staining. In previous studies the proportion of morphologically classifiable cases ranged from 60 to 80%. 9 10 Furthermore, it is more acceptable to diagnose a case as poorly differentiated rather than risk an incorrect subtype classification. In the latter scenario, an erroneous classification could result in the patient missing out on targeted therapy options and genetic studies that could potentially be beneficial in their treatment.

### Role of Immunohistochemical Markers in Subtyping of NSCLC

In well-differentiated NSCLC, morphology alone is sufficient for subtyping in most of the cases. However, in poorly differentiated NSCLC, subtyping is a challenging task when based on H&E alone. 11 In such cases immunohistochemistry should be used as a powerful adjunctive tool. In the current study 57% of adenocarcinomas and 61% of squamous cell carcinomas were subtyped with the help of IHC. Earlier studies have reported the improvement in diagnostic accuracy from 56 to 87% for adenocarcinomas and 75 to 80% for squamous cell carcinomas with resection specimens as gold standard. In many studies published in literature, various antibodies have been evaluated for their putative role in subclassifying NSCLC. 12 13 14 15 16 17 18 An exhaustive list of IHC markers including TTF1, CK7, and Napsin A for adenocarcinoma and p63, p40, DSC 3, CK ⅚, DSG3, and TRIM 29 for squamous differentiation have been evaluated.

### Diagnostic Performance of Individual Markers

#### TTF1 and Napsin A

In the current study, TTF1 was found to have a sensitivity of 97.6% among poorly differentiated adenocarcinomas, while it was 86.6% in the well-differentiated group. Prior studies have reported TTF1 sensitivity for adenocarcinoma in a range of 75 to 86%. 12 13 14 15 16 17 18 In the well-differentiated adenocarcinomas, four cases which were negative for TTF1 revealed morphological features of mucinous adenocarcinomas. This dropped the sensitivity of TTF1 in well-differentiated adenocarcinomas to 86.6%. If these four cases are excluded sensitivity of TTF1 would have been 100%. Goldstein and Thomas and others have shown mucinous adenocarcinomas to be nonreactive for TTF1 and our study also demonstrates the similar trend. 19 Hence, in mucinous adenocarcinomas TTF1 should be avoided and Napsin A or any other alternative markers should be preferred whenever limited panel is applied.

The reported specificity of TTF1 ranges from 83 to 100%. 12 13 14 15 16 17 18 In this study, specificity of TTF1 was 96.75% for adenocarcinomas. The sensitivity of Napsin A varies over a wide range from 33 to 85% in various studies. 13 14 15 18 Its sensitivity is 90.24% which is lower than TTF1 (97.5% vs. 90.24%). Specificity of Napsin A varies from 83 to 100%. 13 14 15 18 We obtained 81.08% specificity for adenocarcinoma which was lower than TTF1. Turner et al and other studies have reported a higher sensitivity and specificity of Napsin A as compared with TTF1 in subtyping of primary pulmonary adenocarcinoma. 17 In our hands, TTF1 was more sensitive and specific than Napsin A. Other studies have reported similar results regarding sensitivity, but our study differs from prior studies in terms of specificity as we found TTF1 to be more specific than Napsin A. We also encountered technical difficulties like background staining and/or nonspecific nuclear staining with Napsin A. TTF1 being a nuclear stain is easier to interpret. There was no consistent association between tumor differentiation and Napsin A expression in our study which is supported in prior studies as well.

#### p63 and p40

Among the squamous markers, we evaluated p63 and p40 for their accuracy in diagnosing squamous cell carcinomas in lung. Both p63 and p40 were positive in all the squamous cell carcinomas. Thus, both had a sensitivity of 100% each. This is in concordance with most of the studies which have established similar results. 16 The difference between the two markers lies in their specificity. p63 was also expressed in 12.6% (9/71) adenocarcinomas. p63 was expressed in adenocarcinomas irrespective of the tumor grade as in the other study. 16 In contrast, p40 was not expressed in any of the adenocarcinomas. Bishop et al have reported significantly higher specificity of p40 as compared with p63 (69% vs. 97%). 20 p40 showed a specificity of 100% versus 89.13% of p63.

### Limited IHC Panel

An accurate classification of poorly differentiated NSCLC becomes very difficult in small biopsies due to scant tissue and in such circumstances IHC markers is of great help. At the same time, newer protocols incorporate the molecular analysis of the tumor and plan targeted therapy for the patient. Applying an extended panel using three or four antibodies is not advisable because many times no tissue will be left for molecular studies. Thus, this study was aimed at delineating minimal panel for diagnosing NSCLC with the view to conserve optimal specimen for molecular genetics.

Our study demonstrated that using TTF1 and p63, overall diagnostic efficacy was 87% in poorly differentiated NSCLC. Thirteen percent NSCLC cases required additional IHC for further classification. 21

When Napsin A was used instead of TTF1, the sensitivity further dropped to 78.4% for poorly differentiated carcinomas. When Napsin A and p40 were used 90.2% carcinomas could be accurately diagnosed. The combination of TTF1 and p40 as a limited panel was optimal in poorly differentiated carcinomas, where 97.56% adenocarcinomas and 96.6% squamous cell carcinomas could be confidently diagnosed. Using a panel of four IHC, 97% of cases could be subtyped, which is only a marginal increase over a limited panel of TTF1 and p40. Thus, TTF1 and p40 can serve as most useful limited panel for NSCLC subclassification.

## Conclusion

In summary, the role of immunohistochemistry in subtyping NSCLC is clear and essential. However, considering the limited tissue available and the necessity for molecular testing, it is advisable to employ a minimal panel. As a first-line panel, TTF1 and p40 should be utilized. An extended panel is only warranted for a small proportion of cases.
